# The association between sleep and depressive symptoms in US adults: data from the NHANES (2007–2014)

**DOI:** 10.1017/S2045796022000452

**Published:** 2022-09-08

**Authors:** Li Chunnan, Shang Shaomei, Liang Wannian

**Affiliations:** 1 Vanke School of Public Health, Tsinghua University, Beijing, China; 2 Institute for Healthy China, Tsinghua University, Beijing, China; 3 School of Nursing, Peking University, 38 Xueyuan Road, Haidian District, Beijing, 100191, China

**Keywords:** Cross-sectional study, depression, epidemiology, sleep

## Abstract

**Aims:**

To assess the association of sleep factors (sleep duration, trouble sleeping, sleep disorder) and combined sleep behaviours with the risk of clinically relevant depression (CRD).

**Methods:**

A total of 17 859 participants (8806 males and 9053 females) aged 20–79 years from the National Health and Nutrition Examination Survey (NHANES) 2007–2014 waves were included. Sleep duration, trouble sleeping and sleep disorder were asked in the home by trained interviewers using the Computer-Assisted Personal Interviewing (CAPI) system. The combined sleep behaviours were referred to as ‘sleep patterns (healthy, intermediate and poor)’, with a ‘healthy sleep pattern’ defined as sleeping 7–9 h per night with no self-reported trouble sleeping or sleep disorders. And intermediate and poor sleep patterns indicated 1 and 2–3 sleep problems, respectively. Weighted logistic regression was performed to evaluate the association of sleep factors and sleep patterns with the risk of depressive symptoms.

**Results:**

The total prevalence of CRD was 9.5% among the 17 859 participants analysed, with females having almost twice as frequency than males. Compared to normal sleep duration (7–9 h), both short and long sleep duration were linked with a higher risk of CRD (short sleep: OR: 1.66, 95% CI: 1.39–1.98; long sleep: OR: 2.75, 95% CI: 1.93–3.92). The self-reported sleep complaints, whether trouble sleeping or sleep disorder, were significantly related with CRD (trouble sleeping: OR: 3.04, 95% CI: 2.59–3.56; sleep disorder: OR: 1.83, 95% CI: 1.44–2.34). Furthermore, the correlations appeared to be higher for individuals with poor sleep pattern (OR: 5.98, 95% CI: 4.91–7.29).

**Conclusions:**

In this national representative survey, it was shown that there was a dose-response relationship between sleep patterns and CRD.

## Introduction

Major depression is the third leading cause of disease burden worldwide, charactered with limiting psychosocial functions and lowering the quality of life (Malhi and Mann, 2018). From 1990 to 2017, the number of reported instances of depression grew by 49.86% globally, indicating that depression remains a serious public health concern (Liu *et al*., 2020*b*). As a complex mental disorder, depression is considered to be impacted by genetic, environmental and gene-environment interactions (Otte *et al*., 2016), as well as modified by lifestyles (Opie *et al*., 2017; Huang *et al*., 2020; Wong *et al*., 2021)

Aside from the well-known dietary and physical activity factors, several studies have been conducted to examine the impact of sleep in the development of depression, but the results were equivocal. Short sleep duration was associated with depression symptoms in a cross-sectional and prospective manner (Lippman *et al*., 2017). One study from rural America supported that short sleep duration was associated with depressive symptoms, whereas another study from rural China supported the notion that both long and short sleep were related with depression (Chang *et al*., 2012; Mohan *et al*., 2017). A meta-analysis of seven prospective studies comprising 25 271 participants for short sleep duration and 23 663 participants for long sleep duration found that both short and long sleep duration were substantially linked with an elevated risk of depression in adults (Zhai *et al*., 2015). Meanwhile, recent meta-analyses have revealed a substantial link between insomnia and depression (Li *et al*., 2016). An analysis of data from the Wisconsin Sleep Cohort Study reveals a substantial long-term relationship between increasing subjective excessive daytime sleepiness and depression (Plante *et al*., 2017). Moreover, several research studies have investigated the joint effect of multiple sleep issues, and revealed that the combined sleep manners were associated with an increased risk of depression (Fernandez-Mendoza *et al*., 2015; Sun *et al*., 2018; Jiang *et al*., 2020). However, the majority of previous studies focused on the elderly or teenagers, varied in sample size, age group and sleep duration definition. The current study aimed to explore the associations between independent sleep factor, as well as combined sleep behaviours, and the risk of depression in general American adults, ranging from 30 to 79 years, utilising National representative data from the NHANES Study.

## Methods

### Study subjects

The NHANES is a series of cross-sectional, complex, multi-stage surveys, conducted by the Centers for Disease Control and Prevention CDC. The NHANES survey combines interviews and physical examinations. The interview includes questions on demographics, socioeconomics, diet and health. Medical, dental and physiological measures, as well as laboratory tests conducted by highly qualified medical experts, comprise the examination component.

In this cross-sectional study, we examined publicly accessible data from participants aged 20–79 years with complete and reliable information (demographics, dietary and health-related behaviours, body measurements and disease information) gathered between 2007 and 2014 waves.

### Assessment of depressive symptoms

Depression was assessed using the Patient Health Questionnaire (PHQ-9), a nine-item screening instrument that asked questions regarding the frequency of symptoms of depression over the past two weeks. Response categories for the nine-item instrument ‘not at all’, ‘several days’, ‘more than half the days’ and ‘nearly every day’ with points ranging from 0 to 3 (Levis *et al*., 2019). A total score ⩾ 10 was considered to be clinically relevant depression (CRD) according to the fourth edition of the Diagnostic and Statistical Manual of Mental Disorders (DSM-IV) (Kroenke *et al*., 2001; Park and Zarate, 2019).

### Assessment of sleep factors and definition of a sleep pattern

Sleep duration was self-reported by the question ‘How much sleep do you usually get at night on weekdays or workdays?’ The quantity of time recorded was grouped as short (<7 h per night), normal (7–9 h per night) and long (>9 h per night) (Chaput *et al*., 2018). The responses to ‘Have you ever told a doctor or other health professional that you have trouble sleeping?’ and ‘Have you/Has SP ever been told by a doctor or other health professional that you have a sleep disorder?’ were used to assess the trouble sleeping and sleep disorders, respectively. The lower and higher risk sleep factors were classified as 1 and 0 for the above-mentioned sleep behaviours to generate overall sleep scores, ranging from 0 to 3. A sleep score of 0 to 1, 2 or 3 indicated a poor, moderate or healthy sleep pattern, accordingly.

### Assessment of potential covariates

The sample person demographics questions involved in the study were administered in the home by trained interviewers using the Computer-Assisted Personal Interviewing (CAPI) system, as follows: age in years at the exam (20–29, 30–44, 45–59, 60–79 years), gender (male, female), ethnicity (Mexican American, Other Hispanic, non-Hispanic White, non-Hispanic Black, non-Hispanic Asian, other Race-including Multi-Racial), education level (less than 9th grade, 9–11th grade/includes 12th grade with no diploma, high school graduate/GED or equivalent, some college or AA degree, college graduate or above) and marital status (married, living with partner, widowed, divorced, separated, never married). Smoking status was measured by the question ‘Have you smoked at least 100 cigarettes in your entire life?’ and classified as ‘yes’ or ‘no’ based on the replies. The physical activity questionnaire is based on the Global Physical Activity Questionnaire and includes questions related to daily activities, leisure time activities and sedentary activities. The suggested metabolic equivalent (MET) scores of 8 points for vigorous work-related/leisure-time activity, 4 points for moderate work-related/leisure-time activity and walking or bicycling for transportation are used to compute metabolic equivalent. The sedentary time refers to the duration spent sitting in a typical day excluding sleeping. Body measurements were obtained by qualified health technicians in the Mobile Examination Centre. Body mass index (BMI) was calculated as weight in kilograms divided by height in metres squared, and then rounded to one decimal place. We utilised the total nutrient intakes (DR1TOT) consumed during the 24-h period prior to the interview on the first day to calculate the 13 components of the Healthy Eating Index (HEI) 2015 score (range, 0–100), as detailed in the previous study (Krebs-Smith, 2018; Liu *et al*., 2020*a*). Daily alcohol intake was obtained on the first-day dietary recall (DR1TOT), and divided into 0 and >0 gm categories. We computed the comorbidity index based on the 11 self-reported diseases (including: arthritis, congestive heart failure, coronary heart disease, angina pectoris, heart attack, stroke, liver condition, diabetes, solid tumour, leukaemia and lymphoma), as detailed in the online Supplementary Table S1.

### Statistical analysis

The baseline characteristics of the study population were presented as percentages according to CRD and sleep pattern status, respectively. Weighted logistic regression models were used to evaluate the relationship between sleep factors (sleep duration, trouble sleeping and sleep disorder) or sleep patterns, and the risk of CRD. Model 1 adjusted for age and gender. Model 2 further adjusted for race, marriage status, education level, smoke status and alcohol intake. Model 3 further included HEI-2015 index, physical activity, sedentary time, BMI and comorbidity index.

The STATA version 14.0 (Stata Corp LP, College Station, TX, USA) was applied for data analysis. R software 3.5.3 was used to create the forest graphs. A *p* value < 0.05 was regarded as statistically significant.

## Results

### The baseline characteristics of study population

Characteristics of the participants according to CRD status are presented in Table 1. Of the 17 859 subjects (49.3% males and 50.7% females, mean (SD) age 47.3 [16.5] years), the overall prevalence of CRD was 9.5%, with 6.7% and 12.3% being in males and females, respectively (Table 1).

**Table 1.** Characteristics of participants by CRD status

Characteristics	CRD (Col%)		
	No (*n* = 16 154)	Yes (*n* = 1705)	*p* value
Age (years)			<0.001
20–29	3009 (92.0)	260 (8.0)	
30–44	4353 (90.4)	462 (9.6)	
45–59	4094 (87.9)	563 (12.1)	
60–79	4698 (91.8)	420 (8.2)	
Gender			<0.001
Male	8217 (93.3)	589 (6.7)	
Female	7937 (87.7)	1116 (12.3)	
Race			0.824
Non-white^a^	9107 (90.4)	966 (9.6)	
White	7047 (90.5)	739 (9.5)	
Marital status			<0.001
Married/Living with partner	9937 (92.7)	777 (7.3)	
Widowed/Divorced/Separated	3057 (84.4)	563 (15.6)	
Never married	3160 (89.6)	365 (10.4)	
Education level			<0.001
⩽High school	7384 (87.6)	1043 (12.4)	
>High school	8770 (93.0)	662 (7.0)	
Sleep duration (hours)			<0.001
<7	6167 (86.5)	960 (13.5)	
7–9	9659 (93.6)	665 (6.4)	
>9	328 (80.4)	80 (19.6)	
Trouble sleeping			<0.001
No	12 642 (94.3)	765 (5.7)	
Yes	3512 (78.9)	940 (21.1)	
Sleep disorder			<0.001
No	14 997 (92.1)	1295 (7.9)	
Yes	1157 (73.8)	410 (26.2)	
Smoking status^b^			<0.001
No	9086 (93.0)	689 (7.0)	
Yes	7068 (87.4)	1016 (12.6)	
Alcohol intake (gm/day)			<0.001
0	11 985 (89.6)	1395 (10.4)	
>0	4169 (93.1)	310 (6.9)	
HEI-2015 index			<0.001
<60	10 728 (89.1)	1317 (10.9)	
⩾60	5426 (93.3)	388 (6.7)	
Physical activity (MET-h/week)			<0.001
0	4109 (85.9)	673 (14.1)	
0–200	10 800 (92.1)	930 (7.9)	
>200	1245 (92.4)	102 (7.6)	
Sedentary time (hours)			0.045
<8	10 904 (90.8)	1110 (9.2)	
⩾8	5250 (89.8)	595 (10.2)	
BMI (kg/m^2^)			<0.001
<30	10 136 (92.1)	868 (7.9)	
⩾30	6018 (87.8)	837 (12.2)	
Comorbidity index			<0.001
0	10 189 (93.4)	720 (6.6)	
>0	5965 (85.8)	985 (14.2)	

*n*, sample size; CRD, clinically relevant depression; BMI, body mass index; HEI-2015, Healthy Eating Index-2015; MET, metabolic equivalent.

a Non-White: Mexican American, other Hispanic, non-Hispanic Black, other race-including multi-racial.

b Smoke 100 cigarettes (or other tobacco) in entire life.

Participants with a poor sleep pattern appeared to be middle and older age, had higher comorbidity index and obesity trends, with lower education level, and more likely to be females, living alone, physically inactive and more sedentary time, more likely to be heavy smokers, on poor eating quality (Table 2). The prevalence of CRD rises in tandem with the worsening of sleep patterns (Table 2).

**Table 2.** Characteristics of participants by sleep pattern status

Characteristics	Sleep pattern (Col%)			
	Healthy (*n* = 8235)	Intermediate (*n* = 6464)	Poor (*n* = 3160)	*p* value
Age (years)				<0.001
20–29	1764 (21.4)	1198 (18.5)	307 (9.7)	
30–44	2298 (27.9)	1772 (27.4)	745 (23.6)	
45–59	1851 (22.5)	1751 (27.1)	1055 (33.4)	
60–79	2322 (28.2)	1743 (27.0)	1053 (33.3)	
Gender				<0.001
Male	4164 (50.6)	3260 (50.4)	1382 (43.7)	
Female	4071 (49.4)	3204 (49.6)	1778 (56.3)	
Race				<0.001
Non-white^a^	4649 (56.5)	3850 (59.6)	1574 (49.8)	
White	3586 (43.5)	2614 (40.4)	1586 (50.2)	
Marital status				<0.001
Married/Living with partner	5184 (63.0)	3801 (58.8)	1729 (54.7)	
Widowed/Divorced/Separated	1369 (16.6)	1332 (20.6)	919 (29.1)	
Never married	1682 (20.4)	1331 (20.6)	512 (16.2)	
Education level				0.034
⩽High school	3810 (46.3)	3072 (47.5)	1545 (48.9)	
>High school	4425 (53.7)	3392 (52.5)	1615 (51.1)	
Smoking status^b^				<0.001
No	4922 (59.8)	3450 (53.4)	1403 (44.4)	
Yes	3313 (40.2)	3014 (46.6)	1757 (55.6)	
Alcohol intake (gm/day)				<0.001
0	6074 (73.8)	4804 (74.3)	2502 (79.2)	
>0	2161 (26.2)	1660 (25.7)	658 (20.8)	
HEI-2015 index				<0.001
<60	5307 (64.4)	4502 (69.6)	2236 (70.8)	
⩾60	2928 (35.6)	1962 (30.4)	924 (29.2)	
Physical activity (MET-h/week)				<0.001
0	2052 (24.9)	1722 (26.6)	1008 (31.9)	
0–200	5639 (68.5)	4164 (64.4)	1927 (61.0)	
>200	544 (6.6)	578 (8.9)	225 (7.1)	
Sedentary time (hours)				<0.001
<8	5648 (68.6)	4377 (67.7)	1989 (62.9)	
⩾8	2587 (31.4)	2087 (32.3)	1171 (37.1)	
BMI (kg/m^2^)				<0.001
<30	5471 (66.4)	4006 (62.0)	1527 (48.3)	
⩾30	2764 (33.6)	2458 (38.0)	1633 (51.7)	
Comorbidity index				<0.001
0	5669 (68.8)	4022 (62.2)	1218 (38.5)	
>0	2566 (31.2)	2442 (37.8)	1942 (61.5)	
CRD				<0.001
<10	7889 (95.8)	5876 (90.9)	2389 (75.6)	
⩾10	346 (4.2)	588 (9.1)	771 (24.4)	

*n*, sample size; BMI, body mass index; HEI-2015, Healthy Eating Index-2015; MET, metabolic equivalent; CRD, clinically relevant depression.

a Non-White: Mexican American, other Hispanic, non-Hispanic Black, other race-including multi-racial.

b Smoke 100 cigarettes (or other tobacco) in entire life.

### Association between sleep and risk of CRD

**Fig. 1.**
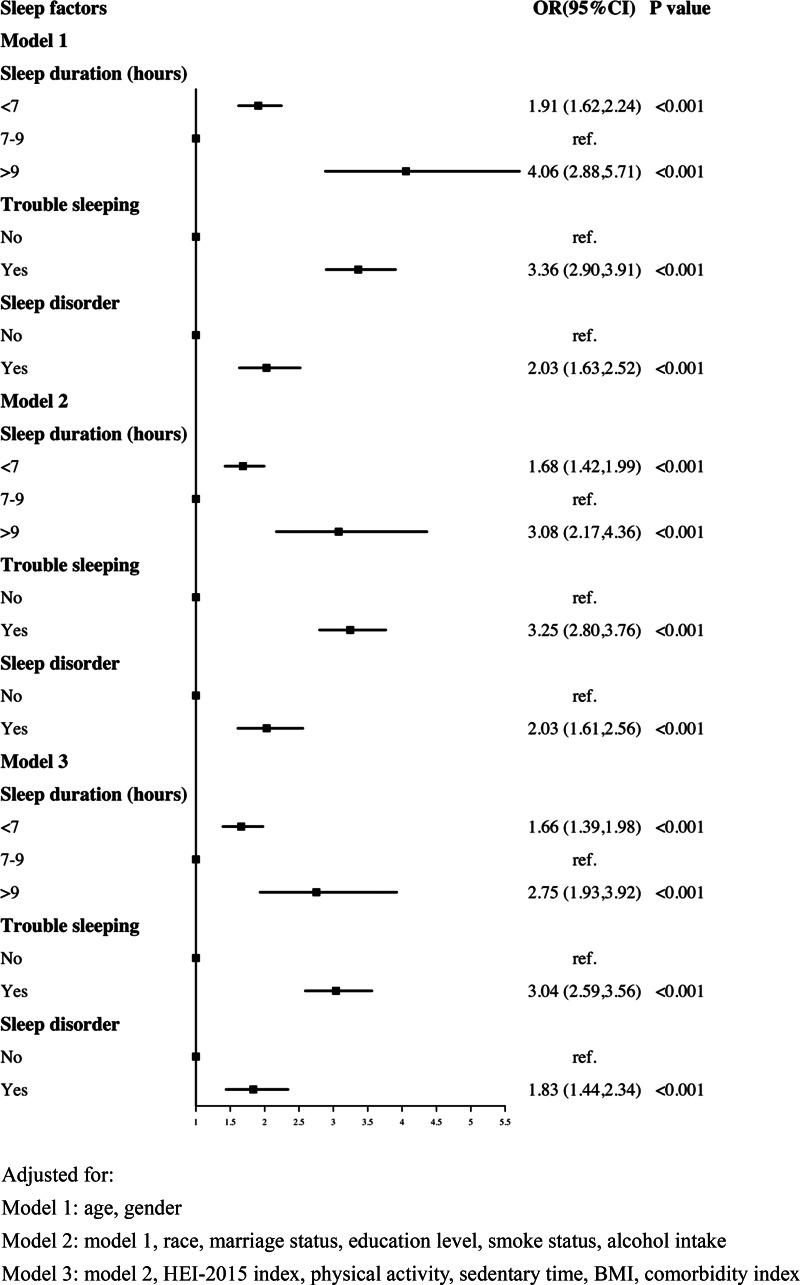
Logistic regression analyses of the association between sleep factors and CRD. Adjusted for: Model 1: age, gender; Model 2: model 1, race, marital status, education level, smoke status, alcohol intake; Model 3: model 2, HEI-2015 index, physical activity, sedentary time, BMI, comorbidity index.

As shown in Fig. 1, in age- and gender-adjusted model (model 1), participants who slept <7 h or >9 h were 1.91 and 4.06 times more likely to have CRD, respectively. Both short (OR: 1.66, 95% CI: 1.39–1.98) and long sleep duration (OR: 2.75, 95% CI: 1.93–3.92) remained significant after adjusting for potential confounding factors (fully adjusted model). Both sleep complaints (including: trouble sleeping and sleep disorder), particularly trouble sleeping (OR: 3.04, 95% CI: 2.59–3.56), was substantially related with CRD as compared to individuals who reported no sleep complaints (fully adjusted model).

**Fig. 2.**
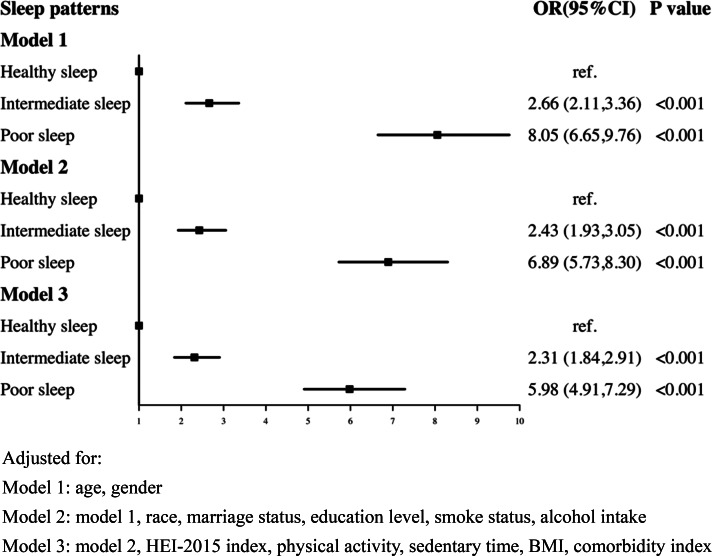
Logistic regression analyses of the association between sleep patterns and CRD. Adjusted for: Model 1: age, gender; Model 2: model 1, race, marital status, education level, smoke status, alcohol intake; Model 3: model 2, HEI-2015 index, physical activity, sedentary time, BMI, comorbidity index.

The relationship of combined sleep factors (including: sleep duration, trouble sleep and sleep disorder) with depression was shown in Fig. 2. Compared to the healthy sleep pattern, participants with poor sleep pattern were associated with a higher possibility of CRD (OR: 5.98, 95% CI: 4.91–7.29) (model 3).

### The association of sleep patterns with risk of CRD by ages

**Fig. 3.**
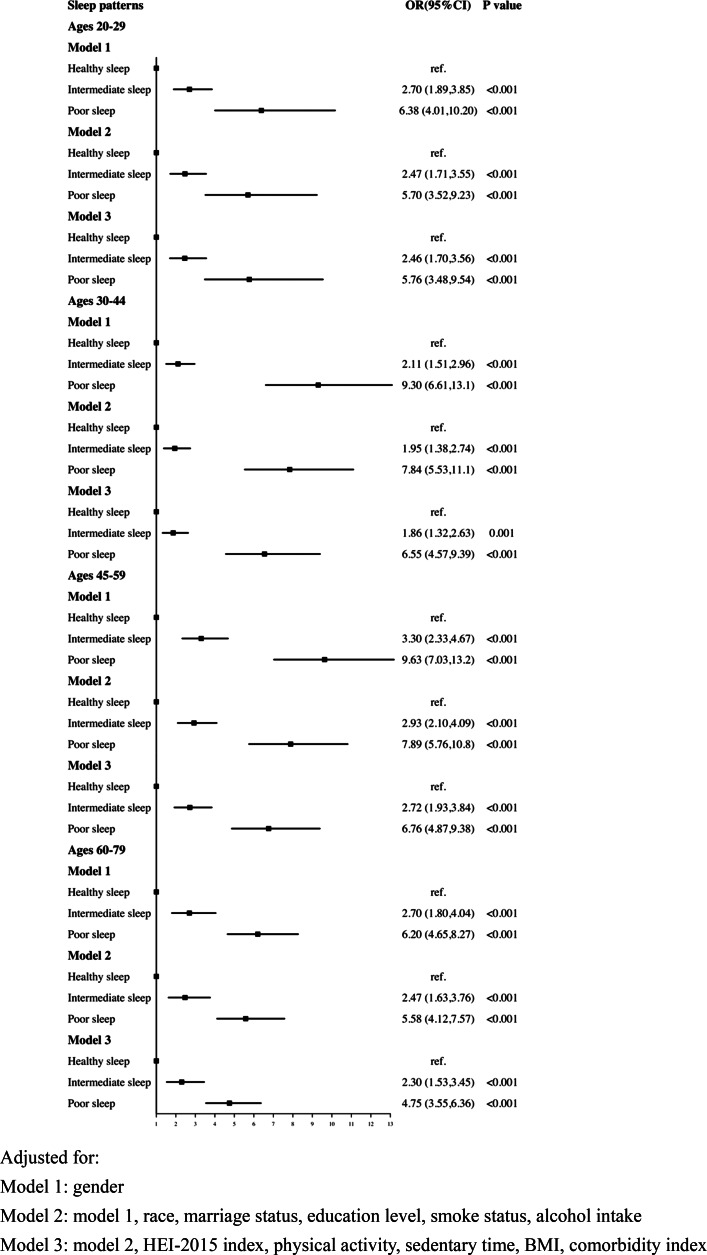
Logistic regression analyses of the association between sleep patterns and CRD stratified by age. Adjusted for: Model 1: gender; Model 2: model 1, race, marital status, education level, smoke status, alcohol intake; Model 3: model 2, HEI-2015 index, physical activity, sedentary time, BMI, comorbidity index.

As shown in Fig. 3, after age stratification, the rising tendency of depression linked with poor sleep pattern remained significant across all age groups, notably in the 30–44 and 45–59 age groups.

## Discussion

To the best of our knowledge, this is the first study conducted on the relationship between sleep behaviours and depression in a large national representative study. We observed that both short and long sleep duration, as well as sleep complaints (trouble sleeping and sleep disorder), were shown to be highly related with CRD. Then we measured the combined associations of sleep duration, trouble sleeping and sleep disorders with the risk of CRD, and participants with a poor sleep pattern had a greater risk of getting depression.

Among the 17 859 participants, the total prevalence of CRD was almost twice as common in females (12.3%) than males (6.7%). This was consistent with earlier findings and was most likely related to individual difference susceptibility and environmental factors (Seedat *et al*., 2009; Kuehner, 2017).

The quantity and quality of sleep are the basic units of good sleep. A recent meta-analysis of six prospective studies from the United States and one from Japan revealed a U-shaped relationship between sleep duration and depression risk (Zhai *et al*., 2015). Another large study, involving over 0.5 million Chinese participants, supported the U-shaped trend as well (Sun *et al*., 2018). This was consistent with our result that, in the fully adjusted model, both short (OR: 1.66, 95% CI: 1.39–1.98) and long sleep duration (OR: 2.75, 95% CI: 1.93–3.92) were associated with depression. However, two other studies merely supported the longitudinal association between short sleep and depression, possibly due to the varied age ranges included (Jackowska and Poole, 2017; Li *et al*., 2017). Aside from sleep quantity, sleep quality is also associated with depression risk (Yang *et al*., 2019; Hu *et al*., 2020). Longitudinal studies suggested that insomnia symptoms or biomarkers measured by polysomnography enhance the incidence of depression by 2.2- to 5.3-fold (Szklo-Coxe *et al*., 2010). And the treatment of insomnia in patients with depression has a positive impact on mood (Gebara *et al*., 2018). Sleep disorders, particularly obstructive sleep apnoea (OSA), are more common in the general population. OSA affects 17% of women and 34% of men in the United States, with a similar prevalence in other countries (Gottlieb *et al*., 2020). Aside from the high prevalence of OSA, patients often fail to report sleep problems to clinicians, resulting in underdiagnosis, which is linked to an increased risk of a variety of negative health outcomes (Jonas *et al*., 2017; Gottlieb *et al*., 2020). For example, a systematic review and meta-analysis of observational studies support the idea that obstructive sleep apnoea may raise the risk of depression (Edwards *et al*., 2020). Furthermore, this study extended on earlier findings by revealing that not only was a single sleep trait associated to depression, but the importance of an overall assessment of sleep factors was emphasised. In all three models, a poor sleep pattern was shown to be associated to the incidence of depression. In accordance with this, a study from rural China has examined sleep behaviours in tandem, indicating that the co-occurrence of sleep duration with objectively sleep complaints is related with the greater risk of depression (Jiang *et al*., 2020). Furthermore, there are significant differences in sleep patterns throughout the lifespan (Hertenstein *et al*., 2018). Previous research has shown that depressive symptoms are more common in older adults with sleep disorders than in young (Cho *et al*., 2008; Paudel *et al*., 2008). A community cohort study of a Chinese population discovered that sleep problems were independently associated with depressive symptoms, particularly among middle-aged and elderly people aged 55–64 (Zhang *et al*., 2021). Similarly, our results supported the association between sleep problems and depression across age groups, particularly the middle-aged population (45–59 years, OR: 6.76, 95% CI: 4.87–9.38).

To date, multiple genome-wide association studies confirmed genetic correlations between sleep duration, insomnia symptoms, excessive daytime sleepiness and depressive symptoms (Hammerschlag *et al*., 2017; Lane *et al*., 2017; Dashti, 2019; Jansen *et al*., 2019; Wang, 2019). A genetically informed twin design revealed that both short (<7 h per night) and long (>9 h per night) sleep enhanced the heritability of depressed symptoms, suggesting that genetic risk for depressive symptoms rises as twins move away from normal sleep duration (7–8.9 h/night) (Watson *et al*., 2014). The underlying mechanism of the link between sleep and depression was unknown, and there were numerous potential processes that contribute to the development of depression through sleep. Sleep and the immune system research studies have revealed that sleep improves immunological defences and that afferent signals from immune cells induce sleep, and immune activation and cytokines may have a role in depression symptoms in some individuals (Dunn *et al*., 2005; Irwin, 2019). Sleep disturbances were independently associated with an increased risk of major depressive disorder, the experimental stimulation of inflammation activates brain regions that control positive and negative effects, and was related with an increase in depressed mood, particularly in women, while antagonism of endogenous inflammation appeared to decrease depressive symptoms (Irwin and Opp, 2017). In addition, chronic sleep deprivation may cause alterations in neurotransmitter receptor systems and neuroendocrine response, contributing to the symptomatology of mental disorders (Novati *et al*., 2008).

Our research was performed using data from a large nationally representative population sample. NHANES's sampling approach ensures that the sample was selected at random and was representative of the whole American population. There were some limitations to the current study. First, as cross-sectional research, we cannot rule out reverse causality due to the nature of the design; Second, the type of sleep disorder is not clearly defined. Finally, all sleep factors were self-reported, which may have recall bias and lack impartiality when compared to sleep monitoring.

## Conclusions

Overall, this study emphasises the independent and combined relationship between sleep-related issues and the risk of depression. Further prospective studies should be conducted to investigate causal or bidirectional relationships between sleep complaints and depression risk. Moreover, it is critical to investigate the genetic association and potential mechanism between sleep complaints and depression, for the effective depression prevention and management.

## Data Availability

The data that support the findings of this study are openly available at https://www.cdc.gov/nchs/nhanes/index.htm, accessed on 15 June 2022.

## Supplementary material

For supplementary material accompanying this paper visit https://doi.org/10.1017/S2045796022000452. click here to view supplementary material
