# Supplementary material for: The association between sleep and depressive symptoms in US adults: data from the NHANES (2007–2014)
Source: Epidemiol Psychiatr Sci. 2022 Sep 8;31:e63. doi: 10.1017/S2045796022000452 (PMC9483824; doi:10.1017/S2045796022000452)
Supplement: Supplementary file 1 [file S2045796022000452sup001.docx]

Table S1 Definition of comorbidity index

| **Variables** | **Definitions** | **Items** | **Included** | **scores** |
| --- | --- | --- | --- | --- |
| MCQ160a | Arthritis | Has a doctor or other health professional ever told you that you had arthritis (ar-thry-tis)? | yes/no | yes:1 no:0 borderline:0 |
| MCQ160b | Congestive heart failure | Has a doctor or other health professional ever told you that you had congestive heart failure? | yes/no |  |
| MCQ160c | Coronary heart disease | Has a doctor or other health professional ever told you that you had coronary (kor-o-nare-ee) heart disease? | yes/no |  |
| MCQ160d | Angina pectoris | Has a doctor or other health professional ever told you that you had angina (an-gi-na), also called angina pectoris? | yes/no |  |
| MCQ160e | Heart attack | Has a doctor or other health professional ever told you that you had a heart attack (also called myocardial infarction (my-o-car-dee-al in-fark-shun))? | yes/no |  |
| MCQ160f | Stroke | Has a doctor or other health professional ever told you that you had a stroke? | yes/no |  |
| MCQ160l | Liver condtion | Has a doctor or other health professional ever told you that you had any kind of liver condition? | yes/no |  |
| DIQ010 | Diabetes | Other than during pregnancy, have you ever been told by a doctor or health professional that you have diabetes or sugar diabetes? | yes/no & borderline |  |
| Mcq220  MCQ230a MCQ230b MCQ230c | Solider tumors /Leukemia/Lymphoma | Have you ever been told by a doctor or other health professional that you had cancer or a malignancy (ma-lig-nan-see) of any kind? | (1) Leukemia; (2) Lymphoma/ Hodgkin's disease (3) Other cancers |  |
|  |  | What kind of cancer was it? |  |  |
